# Development of a highly metastatic model that reveals a crucial role of fibronectin in lung cancer cell migration and invasion

**DOI:** 10.1186/1471-2407-10-364

**Published:** 2010-07-09

**Authors:** Deshui Jia, Mingxia Yan, Xiaomin Wang, Xiangfang Hao, Linhui Liang, Lei Liu, Hanwei Kong, Xianghuo He, Jinjun Li, Ming Yao

**Affiliations:** 1 Laboratory of Experimental Pathology, Shanghai Cancer Institute, Shanghai Jiao Tong University School of Medicine, Shanghai 200032, China; 2 State Key Laboratory of Oncogenes and Related Genes, Shanghai Cancer Institute, Shanghai Jiao Tong University School of Medicine, Shanghai 200032, China

## Abstract

**Background:**

The formation of metastasis is the most common cause of death in patients with lung cancer. A major implement to understand the molecular mechanisms involved in lung cancer metastasis has been the lack of suitable models to address it. In this study, we aimed at establishing a highly metastatic model of human lung cancer and characterizing its metastatic properties and underlying mechanisms.

**Methods:**

The human lung adeno-carcinoma SPC-A-1 cell line was used as parental cells for developing of highly metastatic cells by *in vivo *selection in NOD/SCID mice. After three rounds of selection, a new SPC-A-1sci cell line was established from pulmonary metastatic lesions. Subsequently, the metastatic properties of this cell line were analyzed, including optical imaging of *in vivo *metastasis, immunofluorescence and immunohistochemical analysis of several epithelial mesenchymal transition (EMT) makers and trans-well migration and invasion assays. Finally, the functional roles of fibronectin in the invasive and metastatic potentials of SPC-A-1sci cells were determined by shRNA analysis.

**Results:**

A spontaneously pulmonary metastatic model of human lung adeno-carcinoma was established in NOD/SCID mice, from which a new lung cancer cell line, designated SPC-A-1sci, was isolated. Initially, the highly metastatic behavior of this cell line was validated by optical imaging in mice models. Further analyses showed that this cell line exhibit phenotypic and molecular alterations consistent with EMT. Compared with its parent cell line SPC-A-1, SPC-A-1sci was more aggressive *in vitro*, including increased potentials for cell spreading, migration and invasion. Importantly, fibronectin, a mesenchymal maker of EMT, was found to be highly expressed in SPC-A-1sci cells and down-regulation of it can decrease the *in vitro *and *in vivo *metastatic abilities of this cell line.

**Conclusions:**

We have successfully established a new human lung cancer cell line with highly metastatic potentials, which is subject to EMT and possibly mediated by increased fibronectin expression. This cell line and its reproducible *s.c*. mouse model can further be used to identify underlying mechanisms of lung cancer metastasis.

## Background

According to the World Health Organization, lung cancer is responsible for more than 1.3 billion deaths worldwide annually. Despite advances in the treatment of primary tumours, recurrence and metastasis are the most common cause of death in patients with lung cancer. The current poor understanding of the molecular mechanisms involved in lung cancer metastasis is due, in large part, to the lack of suitable models for its study [1,2]. Although many metastatic models have been successfully used to identify molecular elements during metastasis, most rely on the introduction of tumour cells directly into the systemic circulation. These models do not represent the steps of detachment of tumour cells from the primary tumour--invasion and intravasation--and therefore are unlikely to reveal genes involved in these early steps of metastasis [3,4]. A metastatic model that can represent the full spectrum of metastasis is rare, especially for lung cancer, so it is necessary to develop a spontaneously metastatic model of human lung cancer, so as to provide a platform for uncovering the underlying mechanisms.

EMT, a process whereby cells acquire molecular alterations that facilitate cell motility and invasion, has been shown to play an important role in tumour metastasis [5]. More recently, there are also observations suggesting that the EMT program exists in lung cancer and correlates with the poor prognosis of patients with lung cancer [6,7]. However, these works are mostly based on cultured cell models, and the precise roles of EMT in lung cancer metastasis are still largely unclear. Metastases ultimately develop in secondary organ sites as a consequence of the interactions between tumour cells and the host microenvironment [8]. Fibronectin, a glycoprotein in extracellular matrix and also a mesenchymal maker of EMT, has been implicated in the development of multiple types of human cancer [9,10]. In lung cancer, fibronectin expression is increased and has been implicated in promoting lung cancer growth and conferring resistance to therapy [11,12]. In addition, fibronectin has been shown to promote lung cancer cell migration and invasion by increasing MMP-9 expression or activating FAK signaling [13,14]. However, the specific role and molecular basis of fibronectin in lung cancer metastasis are still elusive.

In the present report, we successfully develop a spontaneously metastatic model of human lung cancer that represents the full spectrum of metastasis, from which a highly metastatic human lung cancer cell line, termed SPC-A-1sci, was derived. This cell line exhibits typical changes in cellular phenotype similar to EMT. Moreover, fibronectin plays an important role in these alterations, and thus resulting in the highly metastatic potentials of this cell line.

## Methods

### Cell lines and cell culture

The human lung adeno-carcinoma cell line SPC-A-1 was obtained from Cellular Institute of Chinese Academy of Science (Shanghai, China). This cell line was originally isolated from the surgical specimens of a Chinese man with advanced lung adeno-carcinoma by Shanghai Chest Hospital and Cellular Institute of Chinese Academy of Science in 1980[15]. The cells were cultured at 37°C under a 5% CO_2 _atmosphere in Dulbecco's Modified Eagle's Medium (DMEM) supplemented with 10% fetal bovine serum (FBS, Hyclone, UT), 100 U/ml penicillin, and 100 μg/ml streptomycin. Cells were regularly certified as free of Mycoplasma contamination.

### Animal experiments

Five- to 6-week-old male congenitally immune-deficient nonobese diabetic/severe combined immune-deficient (NOD/SCID) mice were maintained under specific pathogen-free (SPF) conditions. Mice were manipulated and housed according to protocols approved by the Shanghai Medical Experimental Animal Care Commission. To isolate a highly metastatic cell line, briefly, 2.0 × 10^6 ^of the SPC-A-1 cells were injected subcutaneously (*s.c*) into NOD/SCID mice. When the subcutaneous tumour developed, small pieces of tumour tissue were implanted into the *s.c*. sites of mice in the first generation of mouse models and the primary tumours were excised 4 weeks later. Those mice were sacrificed under deep anesthesia when they showed signs of distress, and visual lung metastases were isolated and *s.c*. implanted into the new recipient mice in the second generation of mouse models for *in vivo *selection. These procedures (lung metastasis, *s.c*. implantation, lung metastasis) were repeated for three rounds. At the end of the selection, the lungs harboring massive metastatic lesions were isolated and *s.c*. implanted into new recipient mice, after which the primary tumour was removed to initiate *in vitro *primary culture.

Subcutaneous tumour implantation was performed as described [15]. In brief, an SPC-A-1 flank-grown tumour was removed from a NOD/SCID mouse and was rinsed and minced in cold phosphate-buffered saline (PBS). The tumour was implanted by the *s.c*. injection of minced tumour tissue suspended in PBS using a 1 cc tuberculin syringe and an 18-gauge needle.

For primary tumour growth assays and spontaneous metastasis *via s.c*. injection, cells (2 × 10^6 ^per mouse) were injected subcutaneously into the right upper flank region of NOD/SCID mice. Mice were monitored weekly for tumour size and evidence of morbidity related to the primary tumour or metastases. Tumour size was quantified in two dimensions using calipers. Tumour volume was calculated as follows: tumour volume (mm^3^) = L × W × W/2, where L represents length and W represents width. Nine weeks later, all mice were sacrificed, and the organs, including lungs and livers, were removed and processed for standard histological studies. For histological analysis, the primary tumours and mouse organs were harvested at necropsy and fixed in 10% formalin. The fixed samples were then embedded in paraffin, and three non-sequential serial sections were obtained per animal. The sections were stained with H&E and analyzed for the presence of metastases.

### Transduction of tumour cells

The GFP-Luc lentiviral vector encoding a fusion gene of GFP and luciferase was generated by inserting the GFP-Luc gene from the plasmid eGFP-2A-CBGr99 (kindly provided by Professor Hammerling) into the *Bam*HI/*Xho*I sites of a pWPXL vector (Addgene). Transductions of SPC-A-1sci and SPC-A-1 cells were performed with the aforementioned lentiviral vector according to instructions supplied by Addgene http://www.addgene.org and stable transfectants were further isolated by cell sorting (Epics Altra, Beckman Coulter) on the basis of their EGFP expression.

### shRNA experiments

The lenti-virus shRNA vector was constructed as described previously [16]. Briefly, Fibronectin and negative control shRNA were subcloned into the *Mlu*I/*Cla*I sites of a pLVTHM vector (Addgene) with the following oligonucleotides respectively: 5'-CGCGTCGGCCCGGTTGTTATGACAATTTttcaagagaAAATTGTCATAACAACCGGGCTTTTTTGGAAT-3'and 5'-CGATTCCAAAAAAGCCCGGTTGTTATGACA ATTTtctcttgaaAAATTGTCATAACAACCGGGCCGA-3' for Fibronectin, and 5'-CGCGTCGTAGCGACTAAACACATCAATTttcaagagaAATTGATGTGTTTAGTCGCTATTTTTTGGAAT-3' and 5'-CGATTCCAAAAAATAGCGACTAAACACAT CAATTtctcttgaaAATTGATGTGTTTAGTCGCTACGA-3' for the negative control. Lenti-virus generation and infection of SPC-A-1sci cells were performed as described above. For experimental metastasis *in vivo*, SPC-A-1sci cells (2 × 10^6 ^per mouse) stably expressing shRNA against fibronectin or negative control were injected into the tail vein of NOD/SCID mice (n = 8). Four weeks later, the mice were sacrificed and the lungs were removed and processed for histological examination.

### Luciferase imaging and GFP imaging

We used a Berthold LB983 NightOwl System (EG&G Berthold, Bad Wildbad, Germany) to monitor the primary tumour growth and distant metastasis of SPC-A-1sci and SPC-A-1 cells in mouse models as previously described [17,18]. For *in vivo *bioluminescence imaging (*in vivo *BLI), the animals were injected i.p. with 150 mg luciferin (Luciferin-EF, Promega) per kg of body weight, anesthetized with pentobarbital (10 mg/ml) in sterile water, and then placed in the NightOwl LB 983 Molecular Light Imager. For *ex vivo *biofluorescence imaging (*ex vivo *BFI), mice lungs were excised after *in vivo *BLI and placed in the chamber of the NightOwl LB 983 Molecular Light Imager and imaged.

### Immunofluorescence and immunohistochemical analysis

The experiments were performed as described previously [19]. For indirect immunofluorescence analysis, cells were plated and grown on glass slides for 18~20 hours and fixed with 4% paraformaldehyde. The slides were then blocked and incubated with the following primary antibodies: Anti-E-cadherin and ZO-1 were obtained from Santa Cruz (1:50), anti-Vimentin from DAKO (1:100), anti-Fibronectin from Abcam (1:100) and anti-α-tubulin and DAPI were obtained from Sigma (1:100). Finally, the slides were incubated with fluorescence conjugated secondary antibody (Sigma) and viewed with a Fluoview FV1000 microscope (Olympus, Japan).

For immunohistochemical analysis, all tissue samples were fixed in phosphate-buffered neutral formalin, embedded in paraffin, and cut into 5-μm-thick serial sections. Immunohistochemical staining with antibodies to E-cadherin (1:50, Santa Cruz), Vimentin (1:25, DAKO) was performed according to standard procedures. Results were observed and photographed with an Axioskop 2 microscope (Carl Zeiss, Oberkochen, Germany) and DP70 Imaging system (Olympus, Japan).

### Soft agar colony formation assay

Colony formation in soft agar was assayed as described previously [20]. Briefly, SPC-A-1sci or SPC-A-1 cells (1000 cells/well) were seeded in 0.3% agar (Sigma) containing culture medium layered on 0.5% agar medium (0.3 ml/well) in 24-well plates and cultured at 37°C under 5% CO_2 _for three weeks. The colonies formed were photographed with an Axioskop 2 microscope (Carl Zeiss, Oberkochen, Germany) and DP70 Imaging system (Olympus, Japan). Pictures of three random fields in each well were obtained from three replicate, and the number of colonies was counted.

### In vitro wound healing assay

The experiment was performed as described previously with little modification [20]. Cells were cultured on Fibronectin-coated or uncoated 24-well plates in complete medium. Upon reaching confluence, the complete medium was replaced with conditioned medium (1% FBS DMEM) for an additional 24 h, and then the cell monolayer was wounded with 200 μL tips. The cells were then cultivated and photographed with a CKX41 microscope (Olympus, Japan) for another 24 h in the conditioned medium. The percent wounded area filled was calculated as follows: {(mean wounded breadth - mean remained breadth)/mean wounded breadth} × 100 (%).

### Migration and invasion assays

Cell migration and invasion assays were performed using 6.5-mm trans-well chambers (8 μm pore size, Corning) as described previously with some modifications [21]. Cells were seeded at 25,000 cells per well into trans-well chambers for migration assays or at 100,000 cells per well into Matrigel-coated trans-well chambers. The wells were washed with PBS after 16 h for migration assays or after 24 h for invasion assays. The cells that had migrated to the basal side of the membrane were fixed and stained with H&E or crystal violet, visualized and photographed with a CKX41 microscope (Olympus, Japan) at 400× magnification and DP20 Imaging system (Olympus, Japan). Pictures of three random fields from three replicate wells were obtained, and the number of cells that had migrated was counted.

### Cell spreading assay

The experiments were performed as previously described [21] with some modifications. Briefly, 24-well plates were pre-coated or uncoated overnight at 4°C with 10 μg/mL Human Fibronectin (Sigma) in PBS. The coated wells were washed with PBS and blocked with 1% BSA in PBS for an additional hour. Aliquots of cells (1.0 × 10^5^) were applied to wells for various time points. After each time point, the wells were washed twice with PBS, and then the adherent cells were visualized and photographed with a CKX41 microscope (Olympus, Japan) at 200 × magnification and DP20 Imaging system (Olympus, Japan).

### Real-time quantitative PCR analysis

Total RNA of cultured SPC-A-1sci and SPC-A-1 cells were isolated using Trizol reagents (Invitrogen) according to the manufacturer's instructions. First-strand cDNA synthesis and amplification were performed using Reverse Transcription Reagents (Takara) following the manufacturer's instructions. Real-time PCR was carried out using a 7300 Real-Time PCR System with SDS RQ Study software (Applied Biosystems) according to the manufacturer's instructions. cDNA templates were combined with SYBR Green premix with Rox (Takara) to perform quantitative-PCR reactions. Primers used for quantitative-PCR were as follows: E-cadherin forward: 5'-TGGCTTCCCTCTTTCATC-3'; E-cadherin reverse: 5'-GTTCCGCTCTGTCTTTGG-3'; Fibronectin forward: 5'-GGAGTTTCCTGAGGGTTT-3'; Fibronectin reverse: 5'-GCAGAAGTGTTTGGGTGA-3'; Vimentin forward: 5'-CTGAACCTGAGGGAAACTAA-3'; Vimentin reverse:5'-AGAAAGGCACTTGAAAGCT-3'; β-actin forward: 5'-AGTGTGACGTGGACATCCGCAAAG-3'; β-actin reverse: 5'-ATCCACATCTGCTGGAAGGTGGAC-3'. Gene expression was normalized to β-actin. All reactions were run in triplicate.

### Immunoblotting assay

Cells were lysed and proteins were detected as described previously [10]. Immunoblotting was carried out with the anti-Fibronectin (Abcam, 1:400) and the β-actin antibody was obtained from Sigma (1:15000).

### Statistics

Data are presented as the means ± SD and were evaluated with Student's *t*-test. P < 0.05 was accepted as statistically significant.

## Results

### *In vivo *selection of highly metastatic human lung cancer cells

**Figure 1:**
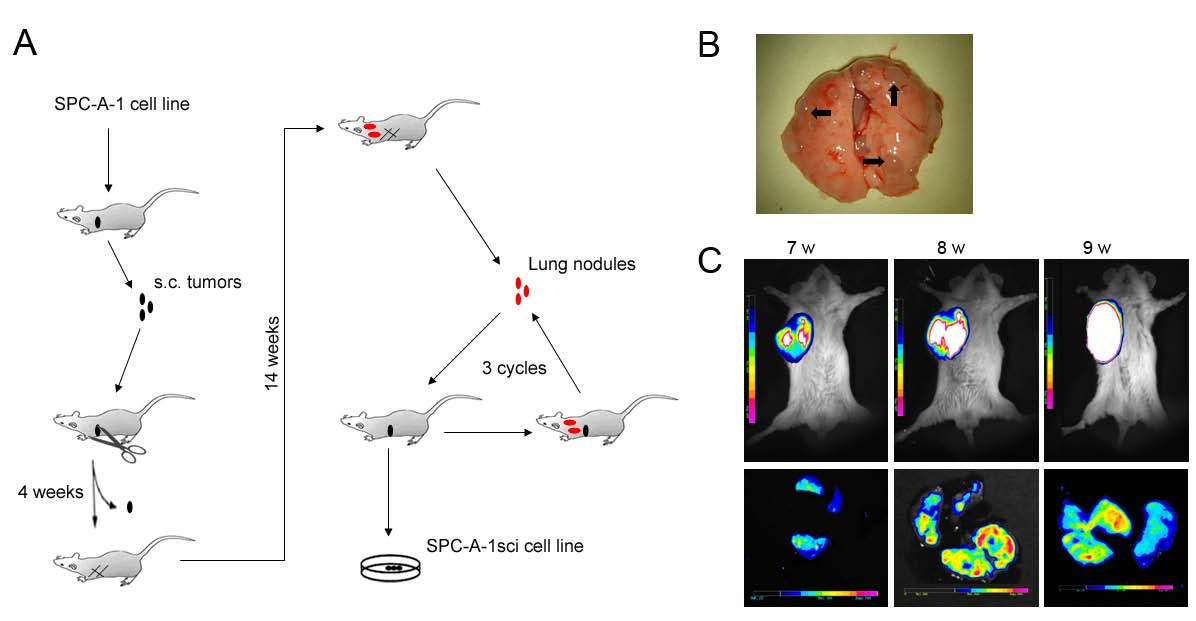
***In vivo *selection of highly metastatic human lung cancer cells**. A, *In vivo *selection scheme. Parental human SPC-A-1 lung cancer cells were subcutaneously injected into NOD/SCID mice. When the subcutaneous tumour developed, small pieces of tumour tissue were implanted into the *s.c*. sites of new recipient mice in the first generation of mouse models, and primary tumours were excised 4 weeks later. Fourteen weeks after resection, visual lung metastases were isolated and re-implanted into the *s.c*. region of new recipient mice for *in vivo *selection. Finally, after three rounds of *in vivo *seleciton, tumour nodules were isolated from the lungs harboring massive metastatic lesions and *s.c*. implanted into new recipient mice, after which the primary tumour was removed to initiate *in vitro *culture and the SPC-A-1sci cell line was derived. B, Representative images of visual inspection of one mouse lungs for the presence of gross tumour nodules (arrows indicate) in the fourth generation. C, Representative bioluminescence images taken from the ventral side of the mice by *in vivo *BLI (upper) for primary tumour growth, and fluorescence images taken from the lungs of one sacrificed mouse *by ex vivo *BFI (lower) for spontaneous metastasis at three sequential time points from week 7 to 9, at 1-week intervals, after *s.c*. injection with GFP-Luc transduced SPC-A-1sci cells.

To develop an advanced metastatic model of lung cancer, we derived a highly metastatic human lung cancer cell line (named SPC-A-1sci) using the poorly metastatic human cell line SPC-A-1 as a starting point (Fig. 1A). The method used is similar to that described previously for generating a metastatic model of breast cancer [22]. Briefly, in order to get pulmonary metastatic nodules for further *in vivo *selection, we removed the *s.c*. primary tumour from tumour-bearing mice to allow sufficient time for distant micrometastases to grow into macrometastases in the first generation of mouse models. Through three rounds of *in vivo *serial selection, the incidence of lung metastasis reached 100% in the fourth generation of the mouse model (Table 1), from which massive metastatic lesions were isolated (Fig. 1B) and *s.c*. implanted into new recipient mice, and the primary tumour was adapted to tissue culture for the generation of the SPC-A-1sci cell line.

**Table 1:** Incidence of pulmonary metastasis in each generation of mouse model during *in vivo *selection

Generation	Macrometastases (mouse numbers)	Micrometastases (mouse numbers)	Selection periods (weeks)
First	2/10	6/10	18
Second	1/7	5/7	14
Third	3/9	7/9	13
Fourth	5/9	9/9	12

To further characterize *in vivo *behaviors of this cell line, we engineered SPC-A-1sci and its parent SPC-A-1 cells with dual reporters of GFP and Luciferase and monitored them in NOD/SCID mice by optical imaging techniques. As shown in Fig. 1C, we could not only detect the primary tumour growth of SPC-A-1sci cells by *in vivo *bioluminescence imaging weekly, but also observe the pulmonary metastases by *ex vivo *fluorescence imaging in one sacrificed animal at week 7 and week 8. Subsequently, numerous metastases could be detected at week 9 in all remaining mice of this group when they displayed signs of being moribund. This was significant compared with the SPC-A-1 group, in which no fluorescence signals of pulmonary metastasis could be monitored at the same end-point when they still displayed an active status (data not shown). Briefly, we successfully developed a highly metastatic cell line from a poorly metastatic lung cancer SPC-A-1 cell line. Whereas the parental line SPC-A-1 required 14 weeks (post-primary tumour resection) for the formation of visible metastatic nodules in lungs, the SPC-A-1sci required only 7 weeks for the formation of large macroscopic nodules in the lungs.

### SPC-A-1sci cells display phenotypic changes consistent with EMT

**Figure 2:**
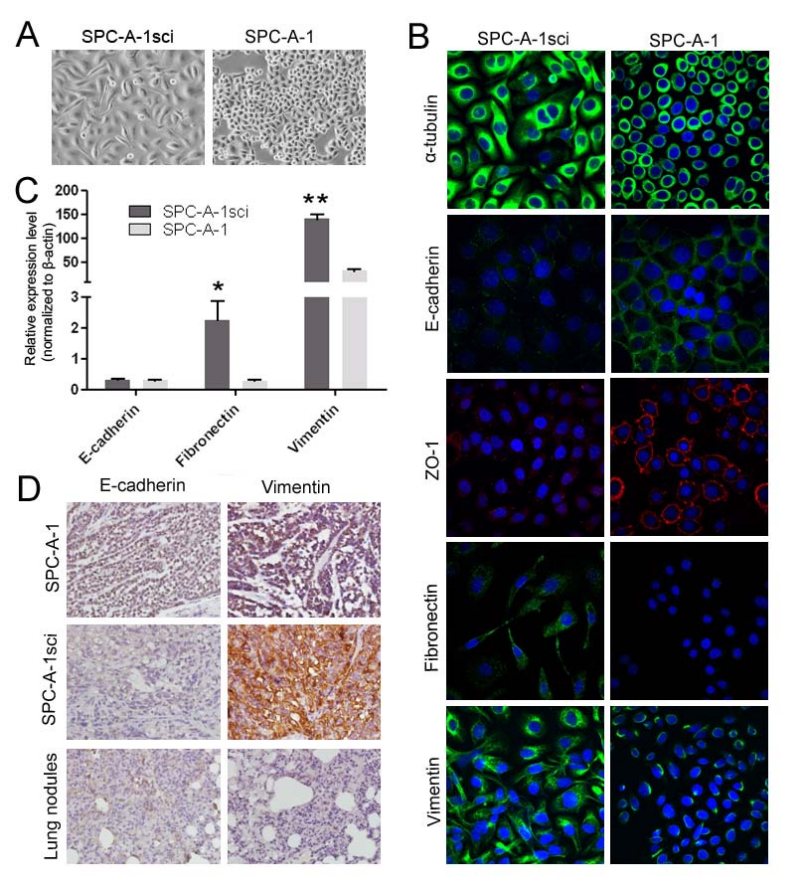
**SPC-A-1sci cells display distinct phenotype consistent with EMT**. A, Representative phase-contrast images of SPC-A-1sci and SPC-A-1 cells are shown. Magnification, 200×. B, Immunofluorescence staining of α-tubulin, E-cadherin, ZO-1, Vimentin and Fibronectin in SPC-A-1sci cells and SPC-A-1cells. The green/red signal represents the staining of the indicated proteins, and the blue signal represents the nuclear DNA staining by DAPI. Magnification, 600×. C, Quantitative-PCR analysis of the mRNA levels of E-cadherin, Vimentin and Fibronectin in SPC-A-1sci and SPC-A-1 cells. Results are expressed as mean ± SD; **, *P *< 0.01; *, *P *< 0.05. D, Expression of E-cadherin and vimentin were examined by immunohistochemical staining of serial sections from *s.c*. primary tumours of SPC-A-1 cells, SPC-A-1sci cells and also pulmonary metastatic nodules of SPC-A-1sci cells. Magnification, 200×.

The generation of motile, invasive carcinoma cells was recently found to share some of the key morphologic and molecular characteristics of EMT [23]. As shown in Fig. 2A, distinct morphological differences between the SPC-A-1sci cell line and its parental SPC-A-1 cell line were observed in cell culture. Whereas SPC-A-1 cells are polygonal and grow in cobblestone clusters, SPC-A-1sci cells are elongated and spindle-shaped, and grow in a scattering pattern to confluence. In addition, the cytoskeleton α-tubulin has also been reorganized in SPC-A-1sci cells, exhibiting the appearance of regular stretching and dispersion by immunofluorescence staining; this is not the case with SPC-A-1 cells (Fig. 2B).

This morphological change is one of the hallmarks of EMT. To determine whether the molecular alterations of EMT occurred in these cells, we examined the localization of several adherent junction proteins, such as E-cadherin and ZO-1, by confocal immunofluorescence staining. The results showed that the two proteins almost disappear from the cell membrane in the SPC-A-1sci cells but show strong membrane staining in their parent SPC-A-1 cells (Fig. 2B). However, the expression levels of E-cadherin have no significant difference between SPC-A-1sci and SPC-A-1 cells by quantitative PCR analysis (Fig. 2C). Furthermore, immunohistochemical staining of *s.c*. primary tumours of SPC-A-1sci cells also confirmed the remarkably reduced expression of E-cadherin (Fig. 2D). In contrast, the expression of mesenchymal markers, including vimentin and fibronectin, whose expression has been shown to positively correlate with the EMT, was strongly increased in SPC-A-1sci cells (Fig. 2B-D).

EMT could be a reversible, dynamic process and may be regulated by the tumour microenvironment, carcinoma cells that have undergone EMT during invasion seem to regain their epithelial characteristics at the metastatic sites [24]. As shown in Fig. 2D, by immunohistochemical analysis, the membrane staining of the epithelial marker E-cadherin significantly increased in the pulmonary metastatic nodules compared to the primary SPC-A-1sci tumour, and otherwise the mesenchymal marker, vimentin, remarkably decreased in the pulmonary metastatic nodules. Hence, both the morphological and molecular changes in SPC-A-1sci cells demonstrated that they had undergone an EMT program.

### SPC-A-1sci cells acquire increased potentials of migration and invasion

**Figure 3:**
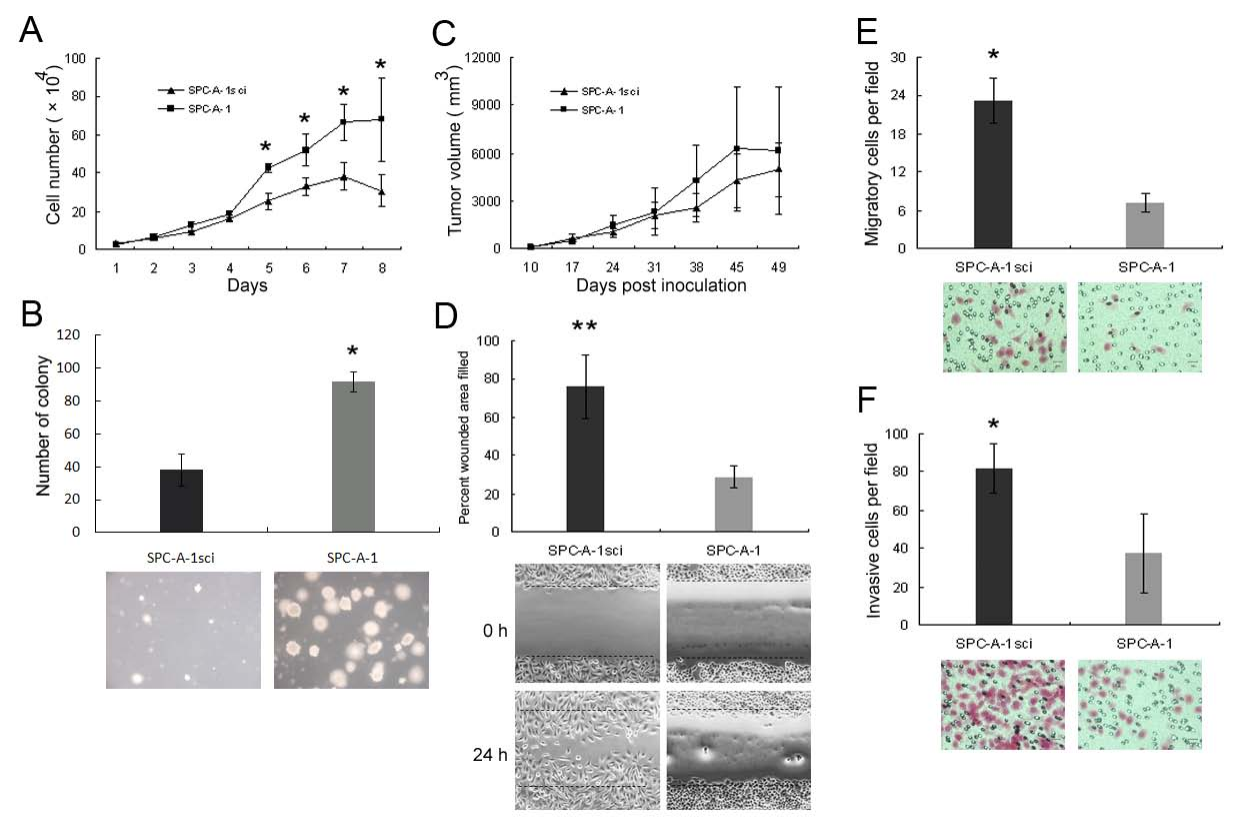
**SPC-A-1sci cells acquire increased potentials of migration and invasion**. A, *In vitro *growth curves of SPC-A-1sci and SPC-A-1 cells, cells (2 × 10^4^/well) were cultured in 24-well plates, after which cells were trypsined and counted at indicated times. Results are expressed as mean ± SD; *, *P *< 0.05. B, Soft agar colony formation of SPC-A-1sci and SPC-A-1 cells, cells (1 × 10^3^/well) were cultured in 24-well plates, colonies were photographed and counted after three weeks. Results are expressed as mean ± SD; *, *P *< 0.05. C, *In vivo *growth curve of SPC-A-1sci and SPC-A-1 cells, cells (2.0 × 10^6 ^per mouse) were *s.c*. implanted into NOD/SCID mice, after which the tumour volume was measured over time. Results are expressed as mean ± SD. C, Motility activities of SPC-A1sci and SPC-A-1 cells were determined by wound healing assays Confluent monolayers of SPC-A1sci and SPC-A-1 cells in fibronectin coated 24-well plates were wounded by scratching, after which the wells were washed, incubated with conditioned medium for 24 h and photographed (lower). Motility was assessed on the basis of percentage of wounded area filled in (upper). Results are expressed as mean ± SD; **, *P *< 0.01. D and E, Trans-well migration and invasion assays of SPC-A-1sci and SPC-A-1 cells For migration assay (D), cells (2.5 × 10^4^/well) were seeded into non-coated trans-well plates and culture for 16 h at 37°C; for invasion assays (E), cells (1.0 × 10^5^/well) were seeded into Matrigel-coated trans-well plates and cultured for 24 h at 37°C, after which cells that had migrated or invaded to the underside of the inserts were stained with H&E and the cells on each insert were photographed (lower) and quantified (upper) at 400× magnification. All experiments were repeated thrice; Results are expressed as mean ± SD; *, *P *< 0.05.

Cell proliferation and colonization are among the necessary functions required for metastatic progression of tumour cells. However, the SPC-A-1sci cells are not more aggressive than the parental population in the proliferation in culture and formation of subcutaneous tumours (Fig. 3A-C). This suggests that the increased metastatic activity of SPC-A-1sci cells does not result from a faster growth rate, but rather from the acquisition of other metastasis-promoting functions.

We next took steps to characterize other cellular properties that might be relevant to metastasis. First, we employed wound healing assays to determine migratory abilities of SPC-A-1sci and SPC-A-1 cells on fibronectin coated or uncoated plates. As shown in Fig. 3D, SPC-A-1sci cells healed the wound dramatically faster than their parent cells did. It's worthy to note that this effect is not substrate dependent, as SPC-A-1sci cells also have an increased migratory ability on non-coated plate (data not shown). Importantly, when seeded on fibronectin-coated plate in this experiment, the poorly metastatic SPC-A-1 cells rapidly spread and show significantly morphologic changes. Subsequently, we also found that SPC-A-1sci cells were more randomly motile than their parent cells by trans-well migration assay (Fig. 3E). Furthermore, we determined the invasive abilities of SPC-A-1sci and SPC-A-1 cells by trans-well invasion assays, and it was found that significantly more SPC-A-1sci cells invaded to the basal side of the membrane than SPC-A-1 cells did (Fig. 3F). These data suggested that the SPC-A-1sci cells acquire enhanced abilities to migrate and invade, which may be associated with their highly metastatic behavior *in vivo*.

### Fibronectin promotes SPC-A-1sci cell invasion and metastasis

**Figure 4:**
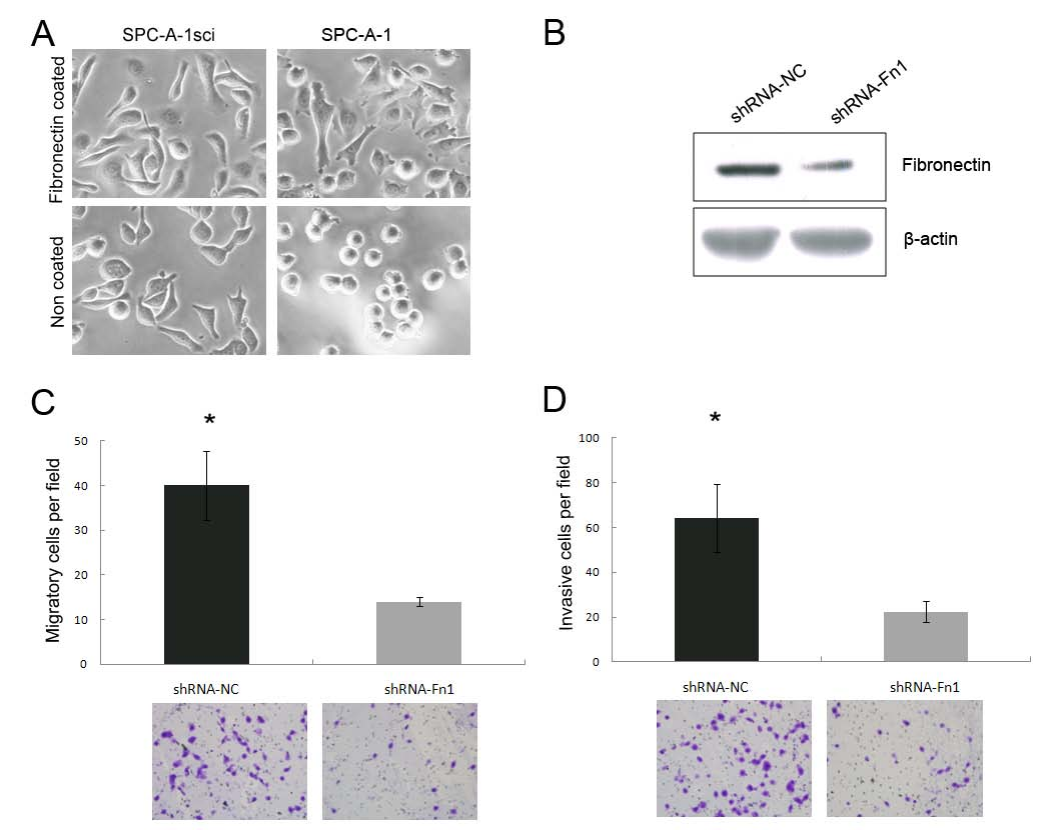
**Fibronectin promotes SPC-A-1sci cell invasion and metastasis**. A, Representative phase-contrast images of SPC-A-1sci and SPC-A-1 cells 4 hours after seeded on fibronectin coated or uncoated plates in cell spreading assays. Magnification, 200×. B, Immunoblotting detection of fibronectin in SPC-A-1sci cells infected with shRNAs against fibronectin (shRNA-Fn1) or negative control (shRNA-NC) as indicated. β-actin was used as a loading control. C and D, Trans-well migration and invasion assays for SPC-A-1sci cells infected with shRNAs against fibronectin (shRNA-Fn1) or negative control (shRNA-NC) as indicated. All experiments were repeated thrice; Results are expressed as mean ± SD; *, *P *< 0.05.

**Figure 5:**
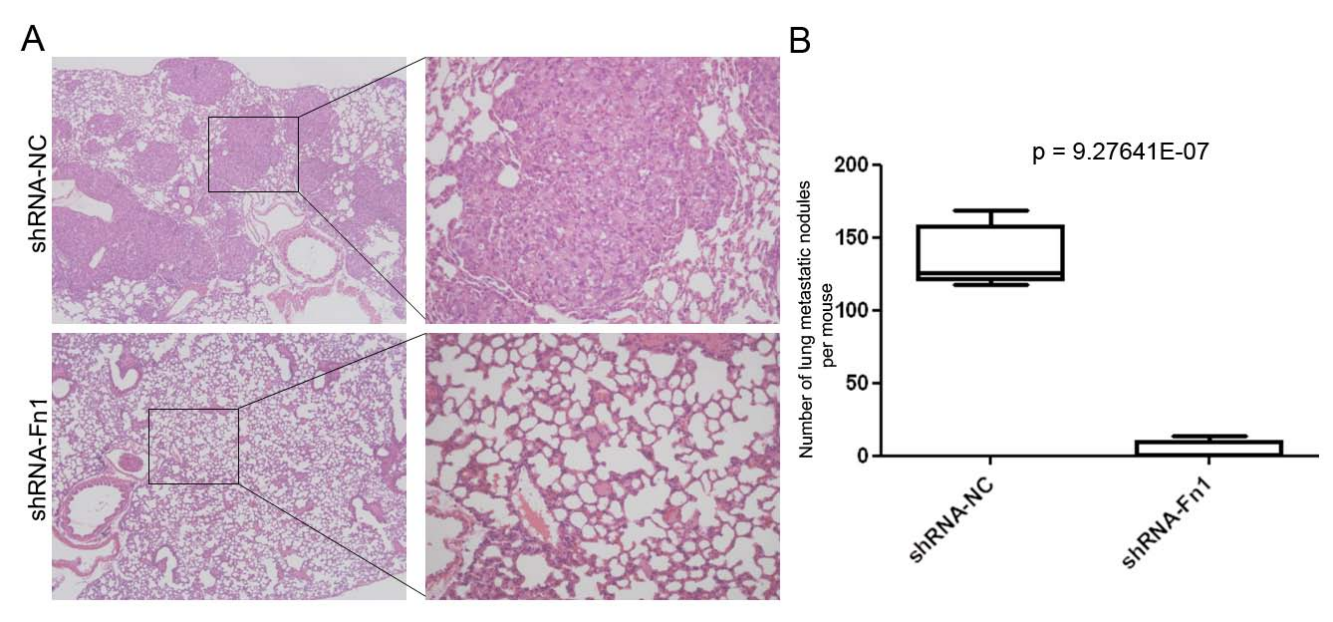
**Knockdown of fibronectin inhibits the metastatic ability of SPC-A-1sci cells *in vivo***. A, Representative images of histological inspection of one mouse lungs for the presence of microscopic lesions (Magnification, 50×; insert, 200×) four weeks after the tail vein injection with SPC-A-1sci cells stably expressing shRNA against negative control (shRNA-NC; Upper) or fibronecetin (shRNA-Fn1; Lower). B, Quantification of microscopic nodules in the lungs of each mouse expressed as mean ± SD of numbers obtained from eight animals in each group (shRNA-NC *versus *shRNA-Fn1); *P *= 9.27641E-07.

As described above, fibronectin could promote the spreading of the parent SPC-A-1 cells. We therefore examined the spreading ability of SPC-A-1sci and SPC-A-1 cells after plating on fibronectin coated or uncoated plates and found that SPC-A-1sci cells spread significantly faster than SPC-A-1 cells did on both conditions (Fig. 4A). Importantly, SPC-A-1 cells spread rapidly and exhibited distinct morphologic changes that are similar to SPC-A-1sci cells when they were cultured on fibronectin coated plates but not on uncoated plates, such as elongated shape and increased migratory protrusions; whereas SPC-A-1sci cells showed little difference between the two conditions and maintained their mesenchymal phenotype. These findings suggested that fibronectin play a crucial role in promoting cytoskeleton reorganization and morphological transition from SPC-A-1 to SPC-A-1sci cells. In order to confirm the direct effects of fibronectin on cell migration and invasion of SPC-A-1sci cells, we used shRNA to knockdown the expression of fibronectin (Fig. 4B) and then measured the migratory and invasive abilities of SPC-A-1sci cells by trans-well assays. The results showed that knockdown of fibronectin significantly decrease the migration and invasion of SPC-A-1sci cells (Fig. 4C-D). Furthermore, the *in vivo *metastasis assays demonstrated the similar results. As shown in Fig. 5, not only the incidence (3/8) but also the number of lung metastatic nodules significantly decreased in mice inoculated with shRNA-Fn1 cells compared to shRNA-NC cells (8/8). These findings suggested a functional role of fibronectin in the SPC-A-1sci cell invasion and metastasis.

## Discussion

Metastasis, the foremost cause of mortality in cancer patients, accounts for more than 90% of all deaths in solid tumour diseases, but the underlying mechanisms remain elusive. Recently, a combination of tumour model systems and microarray profiling technologies has been proved to be effective in identifying relevant genes and pathways for tumour metastasis [4,25]. However, most metastatic models are derived *via *direct injection of cancer cells into the circulation of mice, which puts the focus on end-stage metastatic cells. These models are thus likely to miss the essential genes and pathways required for the early steps of metastasis, including EMT, migration, invasion, and intravasion. It is therefore necessary to develop spontaneous metastatic models that faithfully mimic the selection and evolution of metastasis in human tumours.

Human tumours rarely metastasize from a primary tumour site in immune-deficient mice when they are transplanted into an ectopic site for the particular type of tumour being analyzed. Orthotopic implantation has been proved to be a better approach for the development of metastasis models[26]. Unlike breast cancer and melanoma, however, it is not easy to develop an orthotopic model for lung cancer because of the relative inaccessibility of anatomic location. Fortunately, *s.c*. tumour implantation has been widely used for the majority of human tumour models [27,28], and growing evidence shows that the incidence of metastasis can be greatly promoted by resection of primary tumours in *s.c*. transplantation models [22,29]. It is widely believed that metastatic cells are rare and that they evolve during the late stages of tumour progression from a series of genetic changes that enable the cells to progress through the sequential steps that finally result in growth in distant organ microenvironments [30,31]. Recently, increasing evidence has shown that highly metastatic cells have already existed in their parent cell lines or primary tumours, and the mouse could be used as a "cell sorter" to select for these cells [29,32]. In this study, we successfully isolate a highly metastatic cell line from a poorly metastatic lung cancer SPC-A-1 cell line by *in vivo *selection combined with resection of primary tumours in mice.

Growing evidence suggests that EMT has an important role in tumour metastasis [5,25]. The essential features of EMT are the disruption of intercellular contacts and the enhancement of cell motility, thereby leading to the release of cells from the parent epithelial tissue. The resulting mesenchymal-like phenotype is suitable for migration and thus for tumour invasion and dissemination, allowing metastatic progression to proceed. In this study, we show that SPC-A-1sci cells display morphologic changes consistent with EMT, and that some molecular alterations have taken place in these cells, such as loss of E-cadheirn from the cell membrane of SPC-A-1sci cells. Of note, there is no significant difference in the mRNA level of E-cadherin between SPC-A-1sci and SPC-A-1 cells. E-cadherin mainly binds to plasma membrane to mediate cell-cell interactions, when the membrane binding was destroyed through cleavage by γ-secretase or other proteases[33-35], the functions of E-cadherin would be lost even if the expression levels of it has no changes. Moreover, EMT is a reversible program, and carcinoma cells that have acquired a mesenchymal phenotype can revert to an epithelial state via a MET [36,37]. In the sequential cascade of tumour metastasis, EMT has a positive role in the majority of earlier stages; however, when the disseminating cells arrive in the microenvironment of their targeted organs, the mesenchymal phenotype is not effective in colonizing and growing into macrometastases, such that MET is required in these later stages in order to revert to an epithelial state. Our data show that, to some extent, the expression levels of some EMT markers became switched between the pulmonary metastatic nodules and the primary SPC-A-1sci tumour, as determined by immunohistochemical analysis. Those results indicate that SPC-A-1sci cells undergo EMT, which may be associated with the metastatic potential of SPC-A-1sci cells, including the enhancement of cell motility, invasion, and adhesion. However, a re-differentiation towards an epithelial phenotype, resembling a MET, is also required for the formation of pulmonary metastatic nodules in the end. These data therefore indicate that malignant progression of SPC-A-1sci cells is based on a dynamic process. A large body of literatures indicated that increased cell migration and invasion are functional hallmarks of EMT and are also important steps during the cascade of tumour metastasis [38]. Here, we showed that SPC-A-1sci cells are more aggressive than their parental cells *in vitro*. The potentials of cell motility, migration and invasion are greatly increased in SPC-A-1sci cells. These results suggested that SPC-A-1sci cells acquire the increased abilities to migrate and invade, which further demonstrated that SPC-A-1sci cells have undergone EMT.

The complex interactions between tumour cells and extracellular matrix play important roles in mediating and regulating many processes during tumour metastasis, including cell migration, cytoskeleton reorganization and morphologic transition [39,40]. Fibronectin is an extracellular matrix glycoprotein that plays major roles in cell differentiation, growth and migration, being involved in processes such as wound healing and embryonic development as well as oncogenic transformation [11,41]. In this study, we showed that SPC-A-1sci cells express higher level of fibronectin, and down-regulation of which can significantly decrease both the *in vitro *and *in vivo *metastatic abilities of those cells. Furthermore, fibronectin is also a mesenchymal maker, whose expression positively correlates with EMT [25]. But the contributing roles and precise mechanisms of fibronectin in EMT are still unknown. In this report, we found that fibronectin can induce the poorly metastatic SPC-A-1 cells to take on morphological changes that are similar to SPC-A-1sci cells and also typical of EMT. Briefly, these findings suggested that fibronectin play a crucial role in the highly metastatic behavior of SPC-A-1sci cells.

## Conclusions

In summary, we have successfully established a reproducible *s.c*. mouse model and isolated a highly metastatic human lung adeno-carcinoma cell line by *in vivo *selection in NOD/SCID mice. The new cell line, SPC-A-1sci, possessed highly metastatic potentials and undergone an EMT program, which may be associated with increased expression of fibronectin. To the best of our knowledge, it is the first in the literature that reports the presence of EMT in lung cancer and the functional roles of fibronectin in lung cancer cell invasion and metastasis based on an *in vivo *tumour metastasis model. This model may provide a platform to identify elements that are important in the process of metastasis and to learn how they contribute functionally to the biology of lung cancer metastasis.

## Competing interests

The authors declare that they have no competing interests.

## Authors' contributions

DJ performed major experimental work, and drafted the manuscript. MY, LL, HK carried out the experiments in mice. MW participated in the Trans-well migration and invasion assays. XF performed FACS analysis. LL performed lentiviral transduction of tumour cells. XH helped to draft the manuscript. JL performed the immnuohistochemical experiment and helped to draft the manuscript. MY participated in the design of the study, supervised the laboratory work. All authors read and approved the final manuscript.

## Pre-publication history

The pre-publication history for this paper can be accessed here:

http://www.biomedcentral.com/1471-2407/10/364/prepub

## References

[B1] Gibbons DL Lin W Creighton CJ Zheng S Berel D Yang Y Raso MG Liu DD Lozano G Expression signatures of metastatic capacity in a genetic mouse model of lung adenocarcinoma PLoS ONE 2009 4 4 e5401 10.1371/journal.pone.0005401 19404390 PMC2671160

[B2] Tanaka E Yamashita J Hayashi N Kato S Kondo K Ogawa M A pulmonary metastatic model of human non-small cell lung carcinoma cells that produce a neutrophil elastase-like molecule in severe combined immunodeficiency mice Chest 2003 123 4 1248 1253 10.1378/chest.123.4.1248 12684318

[B3] Clark EA Golub TR Lander ES Hynes RO Genomic analysis of metastasis reveals an essential role for RhoC Nature 2000 406 6795 532 535 10.1038/35020106 10952316

[B4] Minn AJ Gupta GP Siegel PM Bos PD Shu W Giri DD Viale A Olshen AB Gerald WL Massague J Genes that mediate breast cancer metastasis to lung Nature 2005 436 7050 518 524 10.1038/nature03799 16049480 PMC1283098

[B5] Guarino M Rubino B Ballabio G The role of epithelial-mesenchymal transition in cancer pathology Pathology 2007 39 3 305 318 10.1080/00313020701329914 17558857

[B6] Prudkin L Liu DD Ozburn NC Sun M Behrens C Tang X Brown KC Bekele BN Moran C Epithelial-to-mesenchymal transition in the development and progression of adenocarcinoma and squamous cell carcinoma of the lung Mod Pathol 2009 22 5 668 678 10.1038/modpathol.2009.19 19270647 PMC2675657

[B7] Chou TY Chen WC Lee AC Hung SM Shih NY Chen MY Clusterin silencing in human lung adenocarcinoma cells induces a mesenchymal-to-epithelial transition through modulating the ERK/Slug pathway Cellular signalling 2009 21 5 704 711 10.1016/j.cellsig.2009.01.008 19166932

[B8] Joyce JA Pollard JW Microenvironmental regulation of metastasis Nature reviews 2009 9 4 239 252 10.1038/nrc2618 19279573 PMC3251309

[B9] Hu M Carles-Kinch KL Zelinski DP Kinch MS EphA2 induction of fibronectin creates a permissive microenvironment for malignant cells Mol Cancer Res 2004 2 10 533 540 15498927

[B10] Zheng Y Ritzenthaler JD Roman J Han S Nicotine stimulates human lung cancer cell growth by inducing fibronectin expression American journal of respiratory cell and molecular biology 2007 37 6 681 690 10.1165/rcmb.2007-0051OC 17600315

[B11] Ritzenthaler JD Han S Roman J Stimulation of lung carcinoma cell growth by fibronectin-integrin signalling Molecular bioSystems 2008 4 12 1160 1169 10.1039/b800533h 19396378

[B12] Han S Khuri FR Roman J Fibronectin stimulates non-small cell lung carcinoma cell growth through activation of Akt/mammalian target of rapamycin/S6 kinase and inactivation of LKB1/AMP-activated protein kinase signal pathways Cancer research 2006 66 1 315 323 10.1158/0008-5472.CAN-05-2367 16397245

[B13] Han S Ritzenthaler JD Sitaraman SV Roman J Fibronectin increases matrix metalloproteinase 9 expression through activation of c-Fos via extracellular-regulated kinase and phosphatidylinositol 3-kinase pathways in human lung carcinoma cells The Journal of biological chemistry 2006 281 40 29614 29624 10.1074/jbc.M604013200 16882662

[B14] Meng XN Jin Y Yu Y Bai J Liu GY Zhu J Zhao YZ Wang Z Chen F Lee KY Characterisation of fibronectin-mediated FAK signalling pathways in lung cancer cell migration and invasion British journal of cancer 2009 101 2 327 334 10.1038/sj.bjc.6605154 19568240 PMC2720209

[B15] Li Y Tang Y Ye L Liu B Liu K Chen J Xue Q Establishment of a hepatocellular carcinoma cell line with unique metastatic characteristics through in vivo selection and screening for metastasis-related genes through cDNA microarray Journal of cancer research and clinical oncology 2003 129 1 43 51 10.1007/s00432-003-0493-z 12618900 PMC12161897

[B16] Wu D Chen X Guo D Hong Q Fu B Ding R Yu L Hou K Feng Z Zhang X Knockdown of fibronectin induces mitochondria-dependent apoptosis in rat mesangial cells J Am Soc Nephrol 2005 16 3 646 657 10.1681/ASN.2004060445 15677310

[B17] Caceres G Zhu XY Jiao JA Zankina R Aller A Andreotti P Imaging of luciferase and GFP-transfected human tumours in nude mice Luminescence 2003 18 4 218 223 10.1002/bio.729 12950058

[B18] Ciana P Brena A Sparaciari P Bonetti E Di Lorenzo D Maggi A Estrogenic activities in rodent estrogen-free diets Endocrinology 2005 146 12 5144 5150 10.1210/en.2005-0660 16150910

[B19] Yin S Li J Hu C Chen X Yao M Yan M Jiang G Ge C Xie H Wan D CD133 positive hepatocellular carcinoma cells possess high capacity for tumourigenicity International journal of cancer 2007 120 7 1444 1450 10.1002/ijc.22476 17205516

[B20] Takahashi M Furihata M Akimitsu N Watanabe M Kaul S Yumoto N Okada T A highly bone marrow metastatic murine breast cancer model established through in vivo selection exhibits enhanced anchorage-independent growth and cell migration mediated by ICAM-1 Clinical & experimental metastasis 2008 25 5 517 529 10.1007/s10585-008-9163-5 18340424

[B21] Baumann P Cremers N Kroese F Orend G Chiquet-Ehrismann R Uede T Yagita H Sleeman JP CD24 expression causes the acquisition of multiple cellular properties associated with tumour growth and metastasis Cancer research 2005 65 10783 10793 10.1158/0008-5472.CAN-05-0619 16322224

[B22] Munoz R Man S Shaked Y Lee CR Wong J Francia G Kerbel RS Highly efficacious nontoxic preclinical treatment for advanced metastatic breast cancer using combination oral UFT-cyclophosphamide metronomic chemotherapy Cancer research 2006 66 7 3386 3391 10.1158/0008-5472.CAN-05-4411 16585158

[B23] Yang J Weinberg RA Epithelial-mesenchymal transition: at the crossroads of development and tumour metastasis Developmental cell 2008 14 6 818 829 10.1016/j.devcel.2008.05.009 18539112

[B24] Thiery JP Sleeman JP Complex networks orchestrate epithelial-mesenchymal transitions Nat Rev Mol Cell Biol 2006 7 2 131 142 10.1038/nrm1835 16493418

[B25] Yang J Mani SA Donaher JL Ramaswamy S Itzykson RA Come C Savagner P Gitelman I Richardson A Weinberg RA Twist, a master regulator of morphogenesis, plays an essential role in tumour metastasis Cell 2004 117 7 927 939 10.1016/j.cell.2004.06.006 15210113

[B26] Fidler IJ Orthotopic implantation of human colon carcinomas into nude mice provides a valuable model for the biology and therapy of metastasis Cancer metastasis reviews 1991 10 3 229 243 10.1007/BF00050794 1764766

[B27] Sekhon HS London CA Sekhon M Iversen PL Devi GR c-MYC antisense phosphosphorodiamidate morpholino oligomer inhibits lung metastasis in a murine tumour model Lung cancer (Amsterdam, Netherlands) 2008 60 3 347 354 18096271 10.1016/j.lungcan.2007.10.028

[B28] Fork MA Murua Escobar H Soller JT Sterenczak KA Willenbrock S Winkler S Dorsch M Reimann-Berg N Hedrich HJ Bullerdiek J Establishing an in vivo model of canine prostate carcinoma using the new cell line CT1258 BMC cancer 2008 8 240 10.1186/1471-2407-8-240 18706092 PMC2527616

[B29] Cruz-Munoz W Man S Xu P Kerbel RS Development of a preclinical model of spontaneous human melanoma central nervous system metastasis Cancer research 2008 68 12 4500 4505 10.1158/0008-5472.CAN-08-0041 18559492

[B30] Ramaswamy S Ross KN Lander ES Golub TR A molecular signature of metastasis in primary solid tumours Nature genetics 2003 33 1 49 54 10.1038/ng1060 12469122

[B31] van 't Veer LJ Dai H van de Vijver MJ He YD Hart AA Bernards R Friend SH Expression profiling predicts outcome in breast cancer Breast Cancer Res 2003 5 1 57 58 10.1186/bcr562 12559048 PMC154139

[B32] Kang Y Siegel PM Shu W Drobnjak M Kakonen SM Cordon-Cardo C Guise TA Massague J A multigenic program mediating breast cancer metastasis to bone Cancer cell 2003 3 6 537 549 10.1016/S1535-6108(03)00132-6 12842083

[B33] Marambaud P Shioi J Serban G Georgakopoulos A Sarner S Nagy V Baki L Wen P Efthimiopoulos S Shao Z A presenilin-1/gamma-secretase cleavage releases the E-cadherin intracellular domain and regulates disassembly of adherens junctions The EMBO journal 2002 21 8 1948 1956 10.1093/emboj/21.8.1948 11953314 PMC125968

[B34] Najy AJ Day KC Day ML The ectodomain shedding of E-cadherin by ADAM15 supports ErbB receptor activation The Journal of biological chemistry 2008 283 26 18393 18401 10.1074/jbc.M801329200 18434311 PMC2440598

[B35] Noe V Fingleton B Jacobs K Crawford HC Vermeulen S Steelant W Bruyneel E Matrisian LM Mareel M Release of an invasion promoter E-cadherin fragment by matrilysin and stromelysin-1 Journal of cell science 2001 114 Pt 1 111 118 11112695 10.1242/jcs.114.1.111

[B36] Chaffer CL Brennan JP Slavin JL Blick T Thompson EW Williams ED Mesenchymal-to-epithelial transition facilitates bladder cancer metastasis: role of fibroblast growth factor receptor-2 Cancer research 2006 66 23 11271 11278 10.1158/0008-5472.CAN-06-2044 17145872

[B37] Wells A Yates C Shepard CR E-cadherin as an indicator of mesenchymal to epithelial reverting transitions during the metastatic seeding of disseminated carcinomas Clinical & experimental metastasis 2008 25 6 621 628 10.1007/s10585-008-9167-1 PMC2929356 18600305

[B38] Christofori G New signals from the invasive front Nature 2006 441 7092 444 450 10.1038/nature04872 16724056

[B39] Chambers AF Groom AC MacDonald IC Dissemination and growth of cancer cells in metastatic sites Nature reviews 2002 2 8 563 572 10.1038/nrc865 12154349

[B40] Berrier AL Yamada KM Cell-matrix adhesion Journal of cellular physiology 2007 213 3 565 573 10.1002/jcp.21237 17680633

[B41] Pankov R Yamada KM Fibronectin at a glance Journal of cell science 2002 115 Pt 20 3861 3863 10.1242/jcs.00059 12244123

